# Autistic-like Behaviors Associated with a Novel Non-Canonical Splice-Site *DDX3X* Variant: A Case Report of a Rare Clinical Syndrome

**DOI:** 10.3390/brainsci12030390

**Published:** 2022-03-15

**Authors:** Urszula Stefaniak, Roksana Malak, Ewa Mojs, Włodzimierz Samborski

**Affiliations:** 1 Department of Clinical Psychology, Poznań University of Medical Sciences, 60-812 Poznań, Poland; ewa_mojs@poczta.onet.pl; 2 Department and Clinic of Rheumatology, Rehabilitation and Internal Medicine, Poznań University of Medical Sciences, 61-545 Poznań, Poland; rmalak@ump.edu.pl

**Keywords:** *DDX3X*, intellectual and developmental disabilities (IDD), autism spectrum disorders (ASD), rare disease

## Abstract

Background. Heterozygous pathogenic variants in the *DDX3X* gene account for 1–3% of females with intellectual and developmental disabilities (IDD). The clinical presentation is variable, including a wide range of neurological and behavioral deficits and structural defects of the brain. Approximately 52% of affected females remain nonverbal after five years of age. Case presentation: We report a 7 year old nonverbal female with a likely novel de novo pathogenic heterozygous variant in the *DDX3X* gene affecting the non-canonical splice-site in the intron 1 (NM_001356:c.45+12G>A). The patient presents with features typical for the *DDX3X* phenotype, such as: movement disorders, behavioral problems, a diagnosis of autism spectrum disorder (ASD), and some other features uncommon for *DDX3X* such as: muscle hypertonia and spinal asymmetry evaluated through the scoliometer. Conclusions. Due to its rare occurrence, the clinical picture of *DDX3X* syndrome is yet to be fully determined. So far, behavioral disorders, including those from ASD, and neurological abnormalities seem to be the dominant features of this disorder.

## 1. Introduction

*DDX3X* is a ubiquitously expressed ATPase on the X chromosome, *DDX3X*. The mutations of *DDX3X* are known to implicate diseases such as viral infections, inflammation, as well as intellectual and developmental disabilities (IDD) [1,2]. Mutations in *DDX3X* may participate in autism-spectrum disorders (ASD), mostly in females [3]. Referring to the Diagnostic and Statistical Manual of Mental Disorders (DSM-V), ASD mostly affects communication and behavior [4]. Nowadays, the prevalence of ASD is increasing [5]. Worldwide, up to 769 cases of *DDX3X* gene-specific conditions related to ASD have been identified, specifically in 38 males and 731 females across 50 countries [6]. Predominantly prevalent in females, this mutation is inherited in an X-linked dominant pattern and is seldom inherited in males in an X-linked recessive pattern [7]. About 1–3% of females with unexplained intellectual and developmental disabilities IDD carry de novo pathogenic variants in the *DDX3X* gene [7]. Patients present varying degrees of symptoms typical for IDD, including epilepsy, behavior typical for children with autism spectrum disorder, sensory deficits, delays in motor development, microcephaly, and muscle hypotonia. The most common brain malformations can be found in the corpus callosum hypoplasia, polymicrogyria (PMG), and ventricular enlargement [8]. Facial dysmorphism is the phenotypic feature that usually leads parents and caregivers to seek an alternative diagnosis.

Nowadays, pathogenic *DDX3X* variants are predominantly detected in females. Only a few de novo *DDX3X* mutations have been identified in the male population [9]. The *DDX3X* gene located on the X chromosome encodes a conserved DEAD-box RNA-helicase [10]. This gene plays an important role in a variety of fundamental cellular processes including transcription, splicing, RNA transport and translation [7].

We report a seven year old girl with ASD features, delayed psychomotor development, lack of speech, limited and repetitive patterns of behavior, celiac disease, and sensory integration (SI) processing problems with a novel de novo *DDX3X* variant c.45+12G>A. We would like to present our findings because in previous research we did not find enough information about the musculoskeletal system or sensory integration processing disorders. There is a study by Blok et al., (2015) that showed that females with *DDX3X* presented joint laxity and hyperactivity [7]. Even though previous papers presented information about the musculoskeletal system and IDD, we did not find any study that showed how we could measure it. Therefore, we think that the present article may be valid for clinicians, therapists, as well as for parents who would like to improve the intellectual and motor condition of their children with *DDX3X*. 

## 2. Materials and Methods

The patient, who is currently 7 years old, was the first-born child. Her parents did not have any other children. There were no reported miscarriages before or after giving birth. In reference to the interview with the patient’s parents, there were no genetic conditions in this family, children with IDD beyond the second generation, or a diagnosis of epilepsy. The patient was born at 40 + 4 gestational weeks, as a result of an uneventful pregnancy, characterized by regular growth, morphology, and fetal movements. She was born via spontaneous vertex delivery. As a result of a nuchal cord, she was silent and apneic at birth, however engaged in independent respiration following the removal of the umbilical cord and aspiration of the amniotic fluid. Her clinical features are reported in Table 1. Her birth weight was 3680 g, and she had a body length of 54 cm, an occipitofrontal circumference of 34 cm and Apgar scores of 8 at the 5th and 9 at the10th minute of life. A hearing test was conducted and did not show any abnormalities. The same test was repeated at 3 years of age, and again failed to show any shortcomings.

In the first twelve months of life, the patient’s psychomotor development was normal according to her age. An evaluation at three years of age revealed behaviors related to ASD, such as stereotypy, problems with imitation movements that suggested problems with mirror neurons, and dysfunction with sensory processing: tactile defensiveness, lack of speech, and occasional sleep disturbances. Simultaneously, behaviors such as cognitive curiosity, a strong willingness to spontaneously explore the environment, a cheerful disposition and personality, and a fascination with water did not align with features typical of ASD. However, this patient started to attend a kindergarten for children with ASD. A fascination with water transformed into obsessive behaviors with water such as taking off clothing and putting them in the sink. In addition, growth parameters were considered normal, and the patient was slightly taller and heavier than other females of the same age in the normative population. At the age of 3 years and 2 months, she weighed 20,300 g (90th percentile) and was 106 cm tall (50th percentile). At the age of four, she was diagnosed with celiac disease, which confirmed the cause of her severe constipation in earlier years.

**Figure 1:**
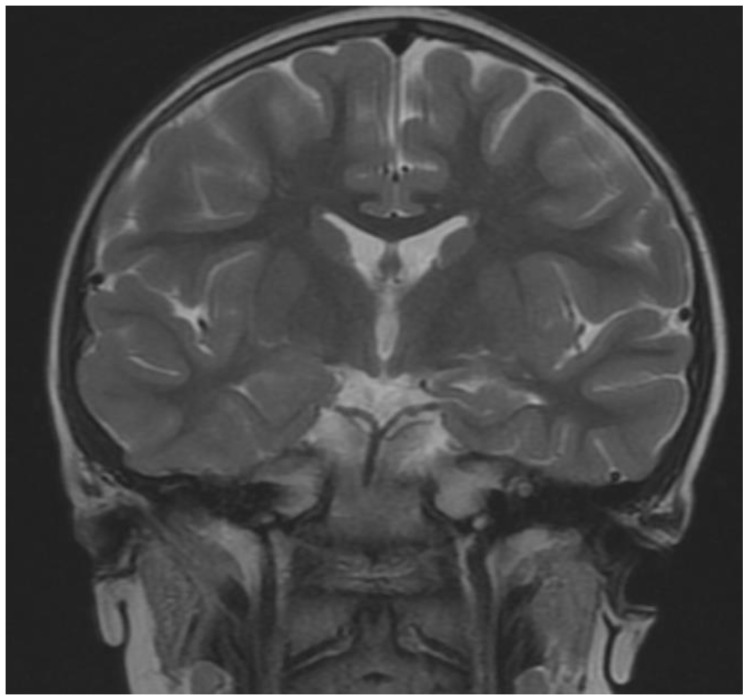
Coronal B (T2-weighted sequence)—Thicker cortical layer on the right side.

**Figure 2:**
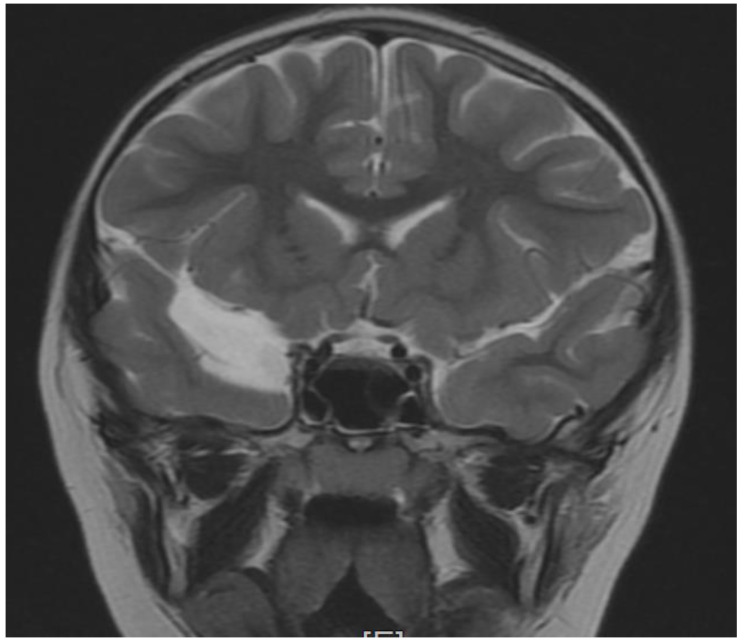
Coronal (T2-weighted sequence)—cyst—at the front of the temporal lobe.

**Figure 3:**
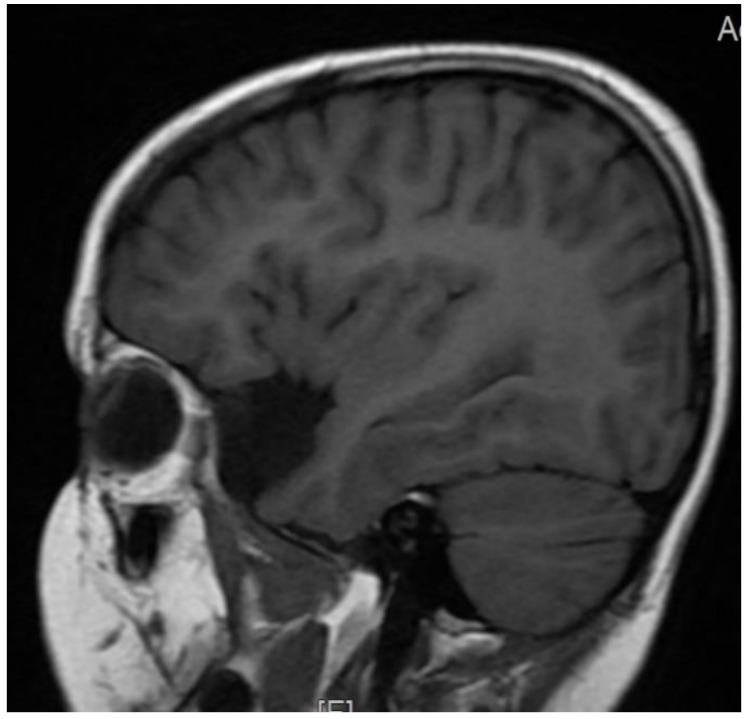
T1-weighted sequence. Cyst modeling temporal lobe.

**Figure 4:**
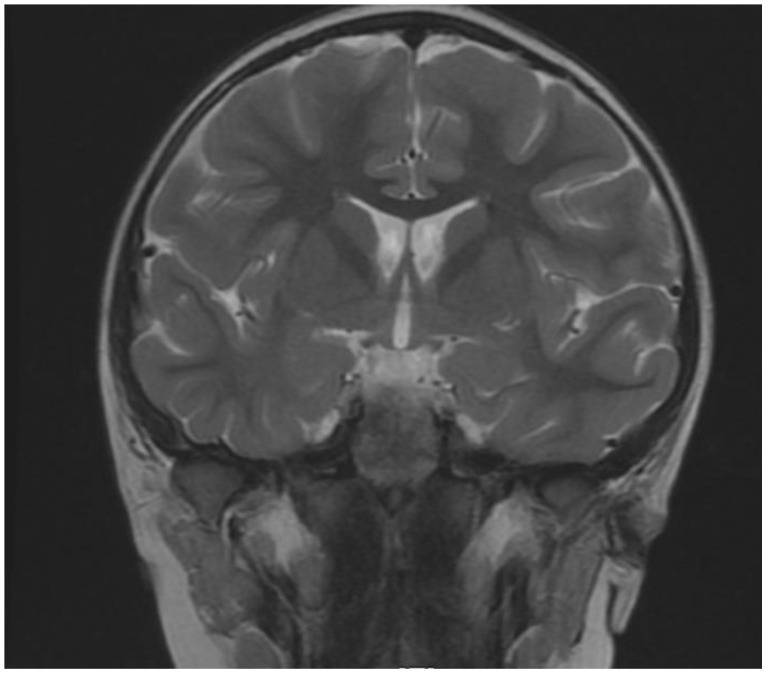
Coronal (T2-weighted sequence). Slight delayed myelination.

The patient’s brain Magnetic Resonance Imaging MRI examination did not reveal any abnormalities in two examinations performed at four and then again at six years of age. In both scans of the middle bottom section of the skull, an arachnoid cyst shaping the front surface of the temporal lobe had been found on the right side (Figure 1, Figure 2 and Figure 3). Moreover, in the right temporal lobe, a slight delayed myelination of white matter and segmented thickness of the cortical layer had been detected (Figure 4). Due to the patient being awake when performing an electroencephalogram (EEG) and not tolerating keeping the electrodes on her scalp due to tactile defensiveness, the few EEGs that were conducted between the ages of four and six were not considered successful. In her fourth year of life, she started to attend sessions with a physiotherapist. During these appointments, she had examinations conducted on her posture, articulations, and motor development. The passive range of motion in hip abduction was measured in a standard supine position by a goniometer (the range of motion was 45 degrees in the right and left hips). The angle of trunk rotation was measured by a scoliometer in the cervical, cervicothoracic (5 degrees), thoracic, and thoracolumbar spines (10 degrees) by a Bunnel scoliometer (C, T, T-L) [11]. The range of motion in the ankle articulation was measured by a goniometer (20 degrees in right, and 25 in left). Motor function was assessed using the International Classification of Functioning, Disability and Health for Children and Youth (ICF-CY). She could walk up and down the stairs d 4508 (the code of ICF-CY), jump d 4503 (the code of ICF-CY), and kick a ball d 4351 (the code of ICF-CY).

Although the child’s motor development progressed, there were some features that did not fit a diagnosis of ASD. Moreover, the behavior of the child started to worsen despite applying strategies for children with ASD. She started to take off her clothing, put water everywhere, and tear paper repetitively. Due to the girl’s curiosity, a desire for social interaction, spontaneous behaviors, and unsuccessful strategies for children with ASD, the neurologist questioned a diagnosis of ASD. Then, a number of genetic tests were performed in search of an underlying cause of the observed phenotype. The karyotype was 46, XX. The results of the PWS/AS MLPA (multiplex ligation-dependent probe amplification) targeted test, the microarray CGH, and the exome sequencing revealed no known pathogenic variants. Moreover, three variants of uncertain clinical significance (VUS) were identified in the HSD17B4 and in *DDX3X* genes.

Exome sequencing has been performed in BluePrint Gentics OY (Espoo, Finland), a CLIA-certified laboratory (#99D2092375), accredited by the College of American Pathologists (CAP#9257331). This test targeted all protein coding exons, exon-intron boundaries (±20 bps) and selected non-coding, deep intronic variants of the genes included in the Clinical Genomics Database and the Developmental Disorders Genotype-Phenotype Database (DD2GP) comprising > 3750 genes. Besides, an analysis for variants that are not located within known clinically associated genes but have properties that make them candidates for potentially disease-causing variants was performed. The median target region coverage was 108 (99.19 sequences covered ≥ 20×). Please see the Blueprint Genetics webpage for further details [12]. Please see Table 2.

**Figure 5:**
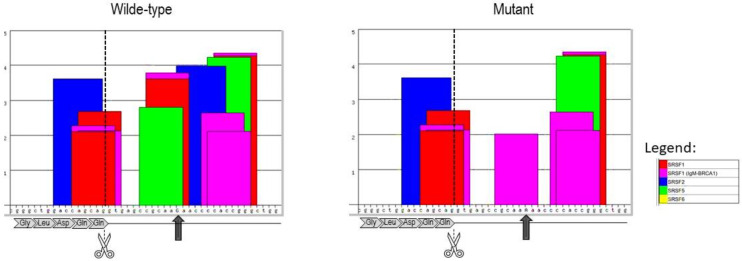
Visualization of the exon-intron junction for exon 1 of the *DDX3X* gene for both the wild-type and mutant genomic sequences (ESE-Finder ver 3.0). The canonical splice-site is shown by seizors; The arrows indicate the variant location. In-silico analyses of the impact on splicing. The substitution at +12 leads to significant alterations in the RNA-recognition motifs of the specific serine/arginine-rich proteins.

Subsequently, segregation studies performed on a maternal DNA sample revealed the maternal origin of the two *HSD17B4* variants. Accordingly, a diagnosis of the autosomal recessive Perrault syndrome was ruled out given their *cis* location. Conversely, the variant in *DDX3X* previously not reported in population databases [13] was found to be absent in the maternal sample and hence likely a de novo event. Even though the paternal sample was not available for co-segregation studies, due to the likely deleterious character of the variant, it is rather improbable that it would originate from the father. Further in-silico analyses suggest its impact on splicing (Figure 5). Experimental verification is currently ongoing.

At three years of age, the patient was presenting with a higher level of arousal and was not adapting well to novel situations. Overall, the child seemed to have severe difficulties with social intelligence and was highly unresponsive to environmental cues at that time [14]. Due to tactile defensiveness, it was extremely difficult to touch the patient [15]. This is why some therapeutic interventions at this point of development could not be implemented. To maintain the ability to have physical contact with the child and to increase the child’s joint attention, proprioceptive and vestibular stimulation were used. Other sensory stimuli were causing “white noise” and destabilized the child [16].

## 3. Results

The Vineland Adaptive Behavior Scales (VABS), Third Edition (Vineland–III) is a standardized assessment that was published in 2016 by Sara S. Sparrow, Domenic V. Cicchetti, and Celine A. Saulnier [17]. This assessment is largely used with children with IDD, and is not only used in the assessment of these children for the development of domain-specific goals for learning but also to determine the overall strengths and weaknesses of these children across several domains. The clinical team used the Vineland-3 Comprehensive Parent/Caregiver Form Report and assessed the following 11 skill domains for the patient: receptive communication, expressive communication, written, personal, domestic, community, interpersonal relationships, play and leisure, coping skills, gross motor skills, and fine motor skills.

For the purposes of this case study, the clinical team chose to report the results of fine and gross motor skills for the patient. While fine motor skills are physical skills that use the small muscles in the hands to manipulate objects, gross motor skills are physical skills that use larger muscles in the arms and legs to carry out movement and coordination.

As can be seen in Table 3, the patient in this study scored v-scale scores for the following skills: 1 for receptive (low), 2 for expressive (low), 4 for written (low), 9 for personal (low), 6 for domestic (low), 6 for community (low), 6 for interpersonal (low), 6 for play and leisure (low), 5 for coping (low), 13 for gross motor (moderately low), and 8 for fine motor skills (low). When interpreting the outcomes of this assessment, the assessor used the v-scale score, with scores of 1–9 indicating low, 10–12 indicating moderately low, 13–17 indicating adequate, 18–20 indicating moderately high, and 21–24 indicating high. Overall, motor skills are considered low for the purposes of the VABS.

Nowadays, thanks to an appropriate genetic diagnosis, behavioral therapy, and appointments with neurologists, the girl has started to have more structure during therapy. She began to attend a kindergarten that is not just for children with ASD but also for children with other dysfunctions. She also started to attend hippo-therapy. Her obsessive behaviors, relating to cutting paper and playing with water, are now rare. She started to have interactions with other children during classes.

## 4. Discussion

Early diagnosis is a very important aspect of treating children, especially those with very rare diseases. An example is the individual described in this case study. The *DDX3X* gene has 69 transcripts (splice variants; ENSEMBL accessed December 2021), which may be the mechanism of regulation of its activity in human tissues, since the encoded proteins vary in their sequence and activity. Alteration of splicing, especially at intron 1, may lead to impairment of the collective network of transcripts and result in the IDD/ASD phenotype.

The patient initially received a diagnosis of ASD as the result of a psychological test at three years of age, and this had an implication in her everyday life. She started to attend a kindergarten for children with ASD, and there her behavior started to regress. It seemed that a psychological diagnosis should be based not only on observation but also on validated tests. An EEG was performed at the age of six, when she was suspected of Rett-like disease or Angelman syndrome due to a fascination with water and possessing blond hair and blue eyes. The girl had the ability to spontaneously explore her environment and be sociable despite presenting with delays in receptive and expressive communication. The DECIPHER database describes the appearance of “cumulative faces” for a patient with *DDX3X*, such as a wide nose bridge and narrow hypoplastic wings. We observe these same characteristics in our patient [7]. The WES test sheds a new light on the case and in this sense cannot be overestimated when it comes to the correct diagnosis of children with autistic-like behaviors.

This is the first study to report a case of a Polish individual with a *DDX3X*-associated disorder. The presence of asymmetry in the spine has not been observed in any previous subjects diagnosed with *DDX3X* syndrome [8]. Changes in the temporal lobe have not yet been detected in previous studies, and that is why we provided MRI scans. The inflammatory cytokine profile is high in IL-1β, IL-6, IL-10 and shows inflammation in the body, and it has not been reported yet in the medical literature or in any *DDX3X* case.

The results of the WES allowed for an end to the diagnostic journey of the presented seven-year-old nonverbal female with delayed psychomotor development. The identified novel variant in the *DDX3X* is most likely affecting the splicing of the gene and leading to an abnormal expression of gene transcripts. The present report emphasizes the clinical utility of exome sequencing in preventing incorrect diagnoses of ASD, cerebral palsy, Rett syndrome, Dandy–Walker syndrome, or an idiopathic diagnosis of IDD. Future observation and treatment should focus on the patient’s speech and will be described and emphasized in a future study.

## 5. Conclusions

In reference to our findings, we suggest a genetic diagnosis for each female with a recent diagnosis of ASD who presents with an atypical phenotype, musculoskeletal problems, pathological changes in MR, and behaviors that do not completely conform to a diagnosis of ASD. Thanks to a proper genetic diagnosis, a proper assessment and treatment can be applied. This enables the proper development of a child with *DDX3X*.

### Limitations of the Study

A limitation of this study is to have described only one female with *DDX3X*. However, it is still a very rare disease.

## Figures and Tables

**Table 1:** Clinical features of the reported individual compared with the characteristics of clinical signs associated with *DDX3X* syndrome.

Clinical Signs in the Presented Case	*DDX3X* Syndrome Clinical Signs
**Development**	
yes	Developmental delay
no	Intellectual disability
yes	Speech delay
**Growth**	
no	Failure to thrive
no	Short stature
no	Microcephaly
yes	Brachycephaly
**Neurologic/behavioral**	
no	Seizures
no	Hypotonia
no	Hypertonia/spasticity
no	Mixed hypo and hypertonia
yes	Sleep disturbance
no	Movement disorders/leg spasticity
yes	Behavior disorders/autism spectrum disorder/aggression
no	Hyperreflexia
**Brain MRI**	
no	Polymicrogyria
no	Corpus callosum hypoplasia/agenesis
no	Ventricular enlargement
no	Key-hole shaped temporal horns
no	Colpocephaly
no	Delayed myelination/decreased cortical white
no	Small pons
no	Small interior vermis
yes	Changes in temporal lobe- cyst
**Sensory**	
no	Vision problems (strabismus, coloboma, astigmatism, nystagmus)
no	Hearing problems
yes	High pain threshold
yes	Temperature dysregulation
**Facial dysmorphism**	
no	Short/down-slanting palpebral fissure
no	Hypertelorism/telecanthus
no	Epicanthal folds
flattened face	Elongated/flattened face/triangular face
high forehead	High/broad forehead
No	Wide nasal bridge/bulbous tip
narrow nose	Short/narrow nose, anteverted nares
no	Micrognathia
no	High arched palate
no	Thin upper lip
no	Low set/protruding/wide ears
no	Smooth/long philtrum
no	Cleft lip/palate
no	Macroglossia
**Other**	
no	Congenital cardiac defects
not applicable	Precocious puberty
no	Feeding difficulties (gastro-esophageal reflux/swallowing)
no	Joint hyperlaxity
yes	Scoliosis/asymmetry in the spine
no	Malformations of the hands
no	Skin pigmentation anomalies
no	Loss/reduced subcutaneous fat
Yes	Higher Inflammatory cytokine profile
yes	IL-1β
yes	IL-6
yes	IL-10

**Table 2:** Characterization of the Variant of Uncertain Significance (VUS) found in the exome sequencing analysis.

Gene	Pos	Transcript	Nomenclature	Consequence	Genotype	Classification
HSD17B4	5:118788281	NM_000414.4	c.11C>G, p(Pro4Arg)	missense_variant	HET	Variant of uncertain significance
	**ID**	**GNOMAD**	**POLYPHEN**	**SIFT**	**MUTTASTER**	
	rs142889209	52/282304	benign	tolerated	polymorphism	
	**OMIM**	**PHENOTYPE**		**INHERITANCE**	**COMMENT**	
		D-bifunctional protein deficiency,		AR		
		Perrault Syndrome				
**GENE**	**POS**	**TRANSCRIPT**	**NOMENCLATURE**	**CONSEQUENCE**	**GENOTYPE**	**CLASSIFICATION**
HSD17B4	5:118813169	NM_001199291.3	c.482A>G, (Glu161Gly)	missense_variant	HET	Variant of uncertain significance
	**ID**	**GNOMAD**	**POLYPHEN**	**SIFT**	**MUTTASTER**	
		0/0	Benign	tolerated	disease causing	
	**OMIM**	**PHENOTYPE**		**INHERITANCE**	**COMMENT**	
		D-bifunctional protein deficiency, Perrault Syndrome		AR		
**GENE**	**POS**	**TRANSCRIPT**	**NOMENCLATURE**	**CONSEQUENCE**	**GENOTYPE**	**CLASSIFICATION**
DDX3X	X:41193562	NM_001356.4	c.45+12G>A	intron_variant	HET	Variant of uncertain significance
	**ID**	**GNOMAD**	**POLYPHEN**	**SIFT**	**MUTTASTER**	
		0/175946	N/A	N/A	N/A	
	**OMIM**	**PHENOTYPE**		**INHERITANCE**	**COMMENT**	
		Mental retardation, X-linked 102		X-linked		

**Table 3:** Results of the Vineland-3 assessment of the patient.

Adaptive Level	Age Equivalent	V-Scale Score/Standard Score	Raw Score	Domain
**Low**	**N/A**	**27**	**N/A**	**Communication**
**Low**	1:01	1	26	Receptive
**Low**	0:11	1	16	Expressive
**Low**	<3:0	4	4	Written
**Low**	**N/A**	**57**	**N/A**	**Daily Living Skills**
**Low**	3:00	9	60	Personal
**Low**	<3:0	6	0	Domestic
**Low**	<3:0	6	6	Community
**Low**	**N/A**	**46**	**N/A**	**Socialization**
**Low**	0:09	6	25	Interpersonal Relationships
**Low**	0:09	5	12	Play and Leisure
**Low**	<2:0	5	6	Coping Skills
**Moderately Low**	**N/A**	**74**	**N/A**	**Motor**
**Moderately Low**	5:00	13	81	Gross Motor Skills
**Low**	2:11	8	37	Fine Motor Skills
**Low**	**N/A**	**49**	**N/A**	**Adaptive Behavior Composite**

## Data Availability

The datasets used and/or analyzed during the current study are available from the corresponding author upon reasonable request. Some data are not publicly available due to containing information that could compromise the privacy of research participants.

## Abbreviations

*DDX3X*	DEAD-Box Helicase 3 X-Linked
IDD	Intellectual and developmental disabilities
DSM-V	Diagnostic and Statistical Manual of Mental Disorders
WES	Whole exome sequencing
MRI	Magnetic resonance imaging
PWS/AS MLPA	Multiplex ligation-dependent probe amplification
EEG	Electroencephalogram
aCGH	Array-Comparative Genomic Hybridization
ASD	Autism Spectrum Disorder
SI	Sensory Integration
ICF-CY	International Classification of Functioning, Disability and Health for Children and Youth
VUS	Variant of Uncertain Significance
